# Case Report: A case of uterine leiomyosarcoma metastasized to the vena cava, excised with the aid of preoperative CT three-dimensional imaging

**DOI:** 10.3389/fonc.2022.905857

**Published:** 2022-08-16

**Authors:** Ling Long, Ling Zhou, Demei Ying, Yan Huang, Juan Yang, Lu Zhou, Sufen Li, Xuan He, Rongkai Xie

**Affiliations:** ^1^ Department of Obstetrics and Gynecology, Xinqiao Hospital, Army Medical University(Third Military Medical University), Chongqing, China; ^2^ Department of Endocrinology, Translational Research Key Laboratory for Diabetes, Xinqiao Hospital, Army Medical University (Third Military Medical University), Chongqing, China; ^3^ Cancer Center, Daping Hospital, Army Medical University (Third Military Medical University), Chongqing, China

**Keywords:** uterine leiomyosarcoma, vena cava, CT 3D imaging, metastases, extracorporeal circulation

## Abstract

Leiomyosarcoma of the uterus (ULMS) is a rare malignant tumor originating from embryonic mesenchymal cells. ULMS tends to metastasize to the lungs, lymph nodes, liver, and bone. Computed tomography three-dimensional (CT 3D) imaging is an advanced diagnostic technique that can track the vessels and their relationships with tumors and reveal the invasion of vessels, including small vessels, around tumors in any slice. Here, we describe a case in which ULMS extended to the retrohepatic inferior vena cava. To date, no report has described resection of metastatic ULMS of the vena cava through supplemental CT 3D imaging. Our patient presented with right lumbar abdominal pain as the main symptom. After using CT 3D reconstruction to accurately assess the relationship between the tumor and the surrounding organs and blood vessels before the operation, the operation was successfully completed through multidisciplinary surgical collaboration.

## Introduction

Leiomyosarcoma of the uterus (ULMS) is a rare malignant tumor that originates from embryonic mesenchymal cells and usually has a poor prognosis (1). Studies have shown that these tumors tend to metastasize to the lung, lymph nodes, liver, and bone (2). However, cases of metastasis to distal organs are rarely reported and reviewed (3). Here, we describe a rare case of ULMS extending into the retrohepatic inferior vena cava. The preoperative CT 3D reconstruction method was used to formulate the surgical treatment plan, and the tumor boundary and the relationship between the tumor and the surrounding tissues were defined. It’s the first reported case of using preoperative CT 3D reconstruction of cancer to assist in the surgical treatment of vena cava metastatic ULMS.

## Case presentation

**Figure 1:**
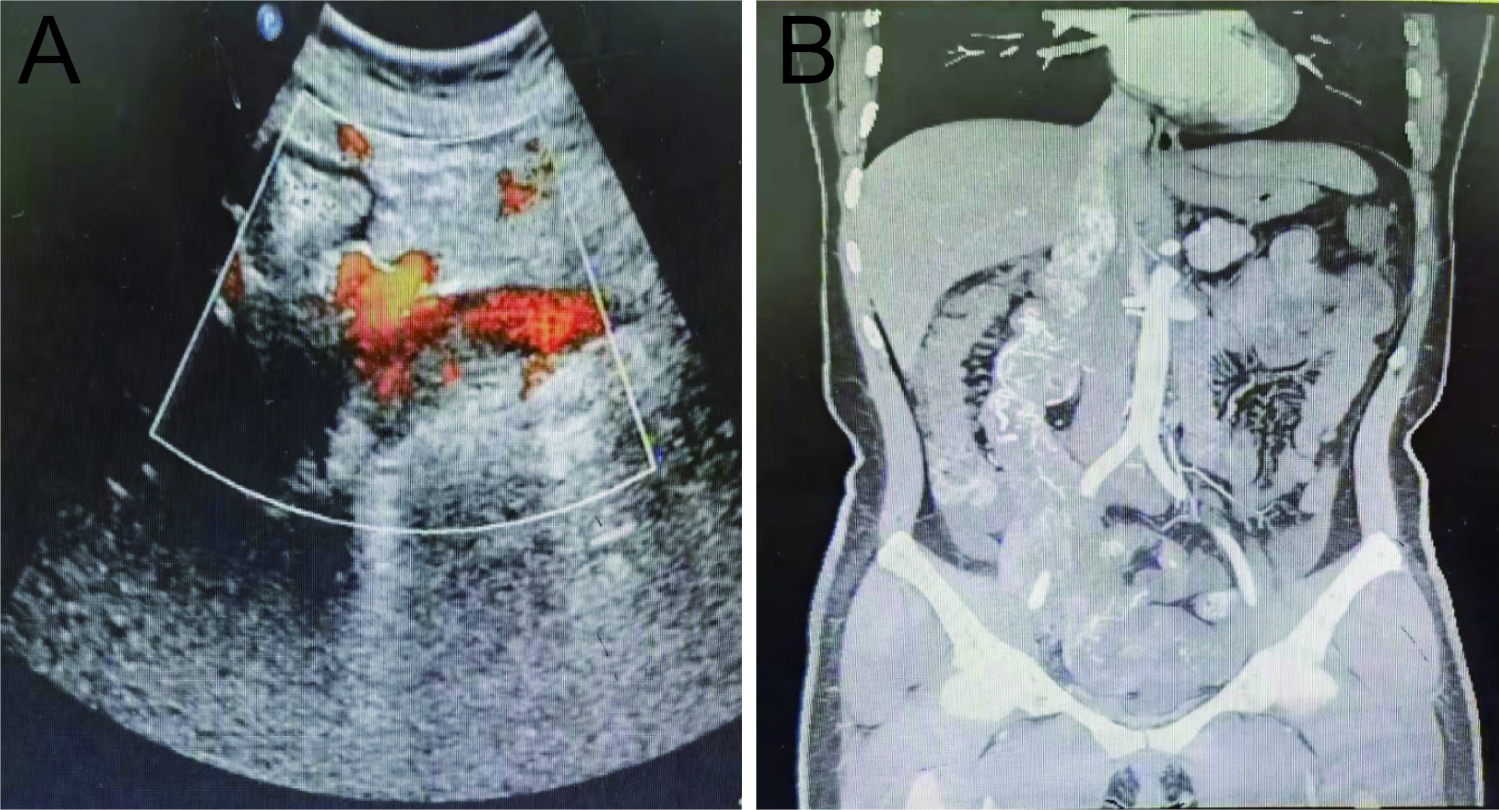
Preoperative pelvic ultrasound and abdominal CTA. **(A)** Preoperative pelvic ultrasound. **(B)** Preoperative abdominal CTA.

A 39-year-old woman was admitted to the hospital on October 18, 2021, with a history of intermittent lumbar abdominal pain for one year, which had worsened in the previous four days. The patient had a history of tolerable right lumbar abdominal pain one year before admission. Six months before admission, an examination in another hospital indicated that her uterine leiomyoma had not been treated. Four days before admission, the patient’s right lumbar abdominal pain was aggravated. After symptomatic treatment in other hospitals, the symptoms were alleviated. On admission, physical examination revealed a flat umbilical mass that was palpable in the lower abdomen. The upper boundary of the mass is level with the umbilicus, and the lower boundary is two fingers below the pubic symphysis. The right border of the mass was unclear, and the left side reached the middle line of the left clavicle and showed poor activity. Gynecological ultrasonography indicated an abnormal uterine shape, a 3.2 mm intrauterine membrane, and a dark fluid area of 64×21 mm in the pelvic cavity. A solid mass, approximately 192×99 mm in size and irregular form, was connected to the right uterine wall and extended upward to the inferior vena cava. Abundant blood flow signals were seen in the parenchyma of the mass (**Figure 1A**). Abdominal computed tomography angiography (CTA) revealed a pelvic soft tissue mass with a focal area of 16×10.5×17.5 cm. The lesion was supplied by bilateral internal iliac artery branches (the right side was dominant). The lesion grew in the right lower abdomen, invaded the main renal vein at the right renal hilum plane, and the inferior vena cava, then reached the hepatic segment of the vena cava (**Figure 1B**). Because of the unclear imaging of the renal vein area, the benign and malignant tumor could not be determined, and the surgical method could not be decided upon. After the second round of hospital consultation, the following were performed: dynamic renal imaging, pelvic mass puncture pathological examination, and abdominal CT 3D imaging.

**Figure 2:**
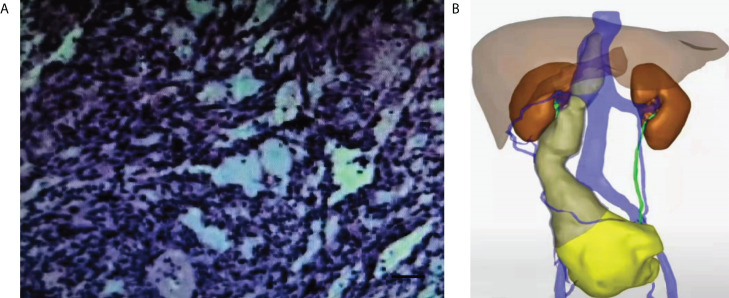
HE staining of pathological tissue and abdominal CT 3D imaging of preoperative pelvic mass puncture. **(A)** HE staining of the pathological tissue of the preoperative pelvic mass (×200). Scale bar, 100μm. **(B)** Preoperative abdominal CT 3D imaging.

Dynamic renal imaging indicated that the total GFR of both kidneys was 102.7 ml/min, a value above the lower normal limit of 82 mL/min; the GFR of the left kidney was 48 mL/min, and the GFR of the right kidney was 54.7 mL/min. Immunohistochemical (IHC) results of pelvic mass punctured biopsy: CD34+, CD117-, dog-1-, Ki-67 20–50%, S-100-, SMA+, Vimentin+, desmin focal+, SDHB+, H-caldesmon+, HMB 45-, MelanA-, ER-, CD10-, CD21, and P16+. *In situ*, hybridization results show EBER-localized myxoid dorsal appearance observed. The Hematoxylin-Eosin (HE) staining of pelvic mass biopsy tissue showed abnormal cell proliferation, deep nuclear staining, and obvious nuclear atypia (**Figure 2A**). Based on the above diagnostic results, the tumor was suggested to be leiomyosarcoma. Abdominal CT 3D reconstruction showed that the lesions grew upward along the right ovarian vein and invaded the inferior vena cava at the right renal hilar plane. The lesions reached the hepatic segment of the vena cava, and a narrowing of the mass was observed before entry into the inferior vena cava. No tumor thrombus was found in the renal vein and lower inferior vena cava (**Figure 2B**).

After the third round of whole-hospital consultation to determine the surgical method and intraoperative plan, the patient underwent total uterine and bilateral adnexectomy, pelvic tumor resection and appendicectomy, greater omentum resection, and inferior vena cava tumor resection under extracorporeal circulation on November 5, 2021. During the operation, the greater omentum widely adhered to the abdominal wall, uterus, bladder, ileum, and colon, and dark red blood-free fluid was observed in the pelvic cavity. After separation of the adhesions, a sizeable pelvic tumor emerged from the right side of the uterus and the adnexal area of the right side and mixed with the uterus. The tumor grows up in the abdominal cavity along the ovarian vein. The right fallopian tube was thickened, and only the right tubal umbrella end could be seen. The tumor was soft in texture and had large blood vessels on its surface which drained into the inferior vena cava behind the duodenum.

The adhesion between the pelvic tumor and loops was isolated. The right adnexa was resected, and the tumor was excised from the pelvic cavity and sent for frozen section analysis. Frozen pathology showed that the pelvic mass was a spindle cell tumor with active hyperplasia, and uterine leiomyosarcoma was considered. Total hysterectomy, left adnexectomy, pelvic tumor resection, appendicectomy, greater omentum resection, and inferior vena cava tumor resection were performed. After resectioning the uterus, the left adnexa, appendix, and greater omentum, residual tumor growth was observed along the right ureter and inferior vena cava and then spread to the upper abdomen. After crossing the hilum of the kidney, the tumor entered the inferior vena cava, thus resulting in apparent thickening, hardening, and loss of vascular elasticity. A urologist separated the tumor from the renal vessels and ureters, and found no palpable renal venous abnormalities. The tumor tissue was not satisfactorily exposed because of the transverse colon and duodenal occlusion. The general surgeon incised the peritoneum on the outside of the duodenum, and dissociated the second and third segments of the duodenum together with the pancreas retro peritoneally. The tumor invaded the inferior vena cava horizontally in the renal vein of the posterior duodenum, and the inferior vena cava was thickened and the mass was palpable. A heart surgeon opened the posterior peritoneum to expose and dissociate the external iliac artery. After heparinization, the external iliac artery was inserted into the extracorporeal circulation artery. The tumor was then extracted from the inferior vena cava, and the laceration of the inferior vena cava was sutured. After the patient’s circulation stabilized, her arterial blood gas results were normal. Pull out the arterial catheter from the external iliac artery and tie the knot simultaneously; Stop the machine. After the absence of tumor residue in the pelvic cavity was confirmed, the abdomen was closed.

**Figure 3:**
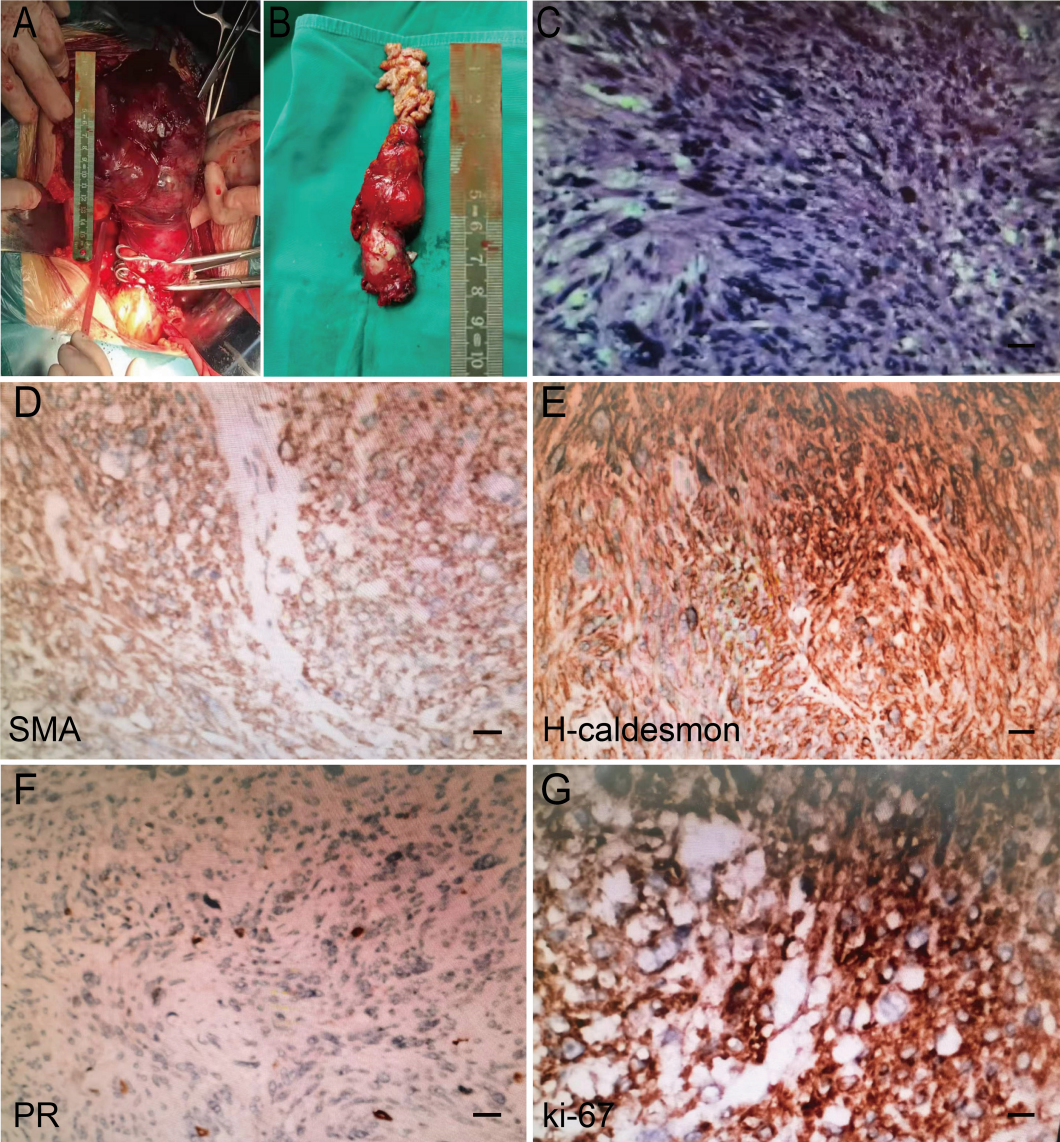
Intraoperative gross specimen and postoperative pathological HE staining results. **(A)** Tumors in the pelvic cavity. **(B)** Tumor in the inferior vena cava. **(C)** HE staining results in postoperative pathological examination (×200). Scale bar, 100μm. **(D-G)** Postoperative pathological IHC staining (SMA, H-Caldlesmin, PR, Ki-67 positive).

**Figure 4:**
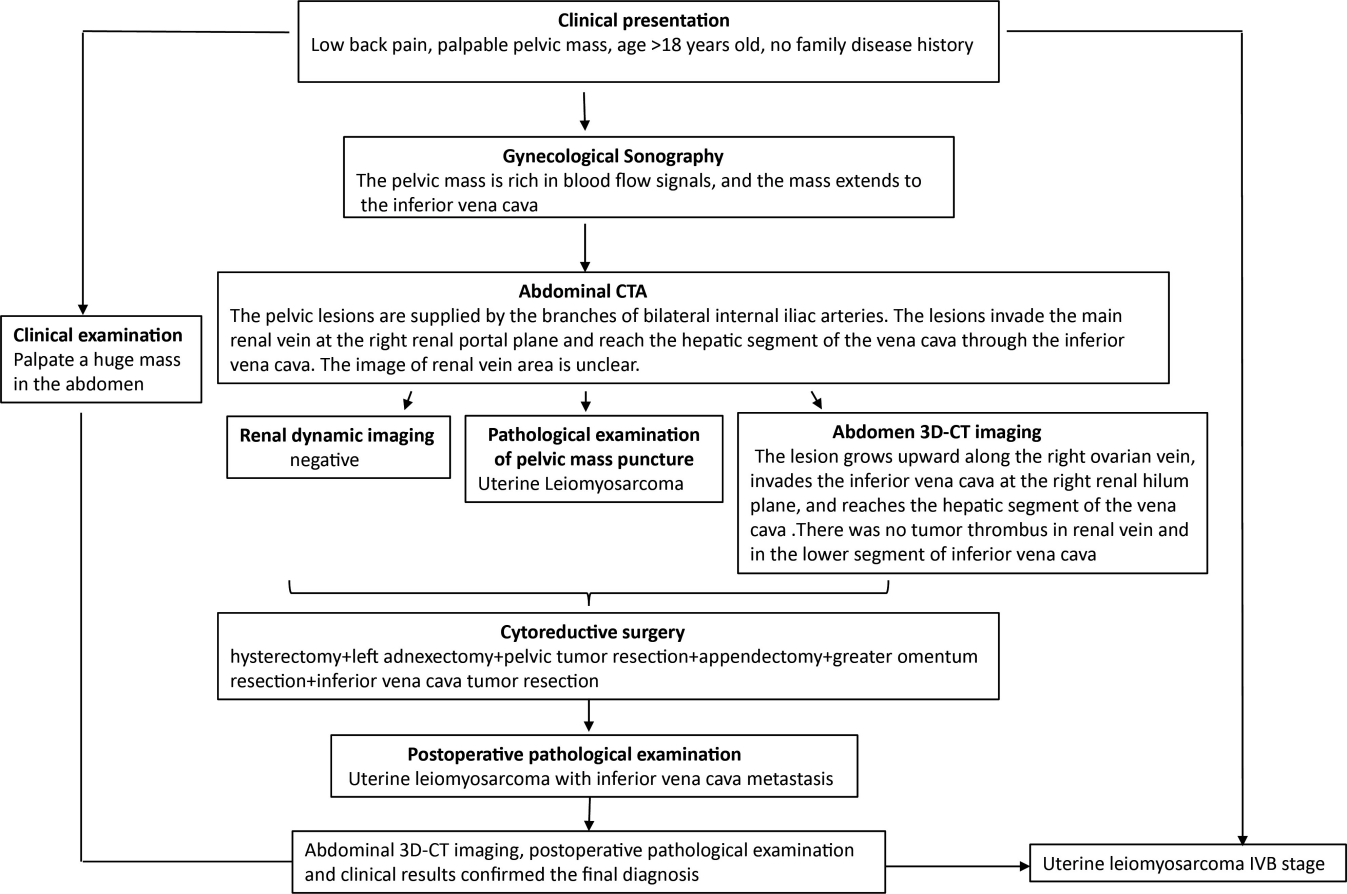
Diagnostic workflow diagram. The clinical manifestations of abdominal and low back pain must be differentiated from various age-related urinary system diseases. The patient underwent clinical examination, gynecological Doppler ultrasound, abdominal CTA, pelvic mass puncture biopsy (HE, IHC), and abdominal CT 3D imaging. Preoperative abdominal CT 3D imaging made the clear scope of the lesion, the tumor was completely removed during the operation, and the final diagnosis was made by postoperative examination.

Postoperative lesions were found to be solid tumors with a size of 16×10.5×17.5 cm (**Figure 3A**). The size of the inferior vena cava tumor was approximately 8×3×2 cm (**Figure 3B**). Postoperative pathological HE staining showed abnormal cell proliferation, dark blue nuclei, disordered arrangement, the disappearance of swirling arrangement, and obvious nuclear atypia (**Figure 3C**). The IHC results revealed desmin+, HMB45-, MelanA-, MDM-2-, SMA+, H-caldesmon+, Ki-67 20–60%, Vimentin+, CD10-, ER-, PR focal+. CD34-, S-100-, and WT-1-, findings consistent with the preoperative biopsy specimen. The specific IHC results are as follows: two important indicators of leiomyosarcoma, SMA and H-Caldlesmin protein, were positive, progesterone receptor (PR) derived from the uterus also showed focal positive, and the Ki-67 index was estimated to be 60% (**Figures 3D–G**). On this basis, ULMS with inferior vena cava metastasis was diagnosed. The final diagnosis: uterus leiomyosarcoma IVB stage. The detailed diagnosis process is shown in **Figure 4**.

**Figure 5:**
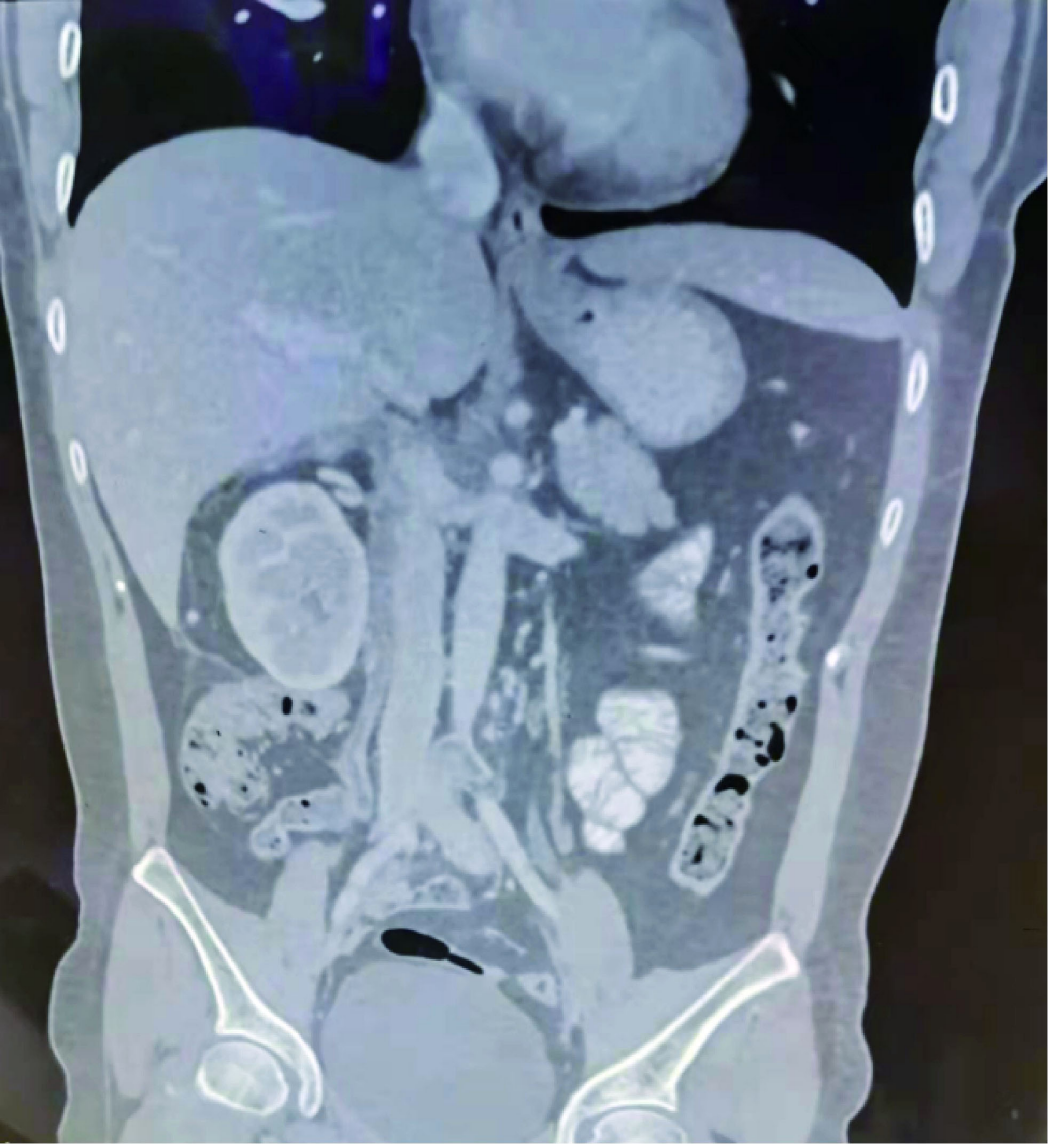
Abdominal CTA 3 months after surgery.

Doxorubicin chemotherapy was administered once after surgery, and the patient was discharged on the 12th postoperative day. Four chemotherapy cycles were administered. CT examinations were performed every three months (**Figure 5**), and the patient was in good condition, with no recurrence or metastasis three months after surgery.

## Discussion and conclusions

ULMS, a rare gynecological tumor accounting for approximately 1% of all uterine malignancies, is the most common subtype of uterine sarcoma, with an annual incidence of 0.64/100,000 (4). The patient’s age at onset is usually 50-55 years old. Although rare, ULMS leads to many deaths because of frequent recurrence and distant metastasis; the 5-year overall survival rate ranges from 57% in stage I to 16% in stage IV (5). The survival rate of women diagnosed with metastatic ULMS is only 10-15% (6). Recurrence rates range from 45 to 75%, and many recurrence sites are found. The most common site of the first recurrence is the lung (40%). Common sites of metastasis include the peritoneal cavity, skin/soft tissue, liver parenchyma, brain, and bone (7). Patients with metastatic tumors usually die within two years.

The most common symptoms of ULMS include abnormal uterine bleeding (56%), abdominal mass or uterine enlargement (54%), and abdominal pain (22%) (8). Because these symptoms are similar to those of other benign uterine tumors in their clinical manifestations, ULMS diagnosis is often delayed.

No laboratory tests or imaging studies can reliably diagnose ULMS. Although some patients have elevated lactate dehydrogenase or CA125 levels, these markers are nonspecific and are not reliable predictors of ULMS. Magnetic resonance imaging remains a good method for the preliminary diagnosis of uterine tumors (9). Typical histological features of ULMS include spindle cells with a blunt terminal nucleus and active mitotic activity (ten mitoses per ten high energy fields). The morphology shows atypical nuclear pleomorphism, cell proliferation with fascicular growth, coagulative necrosis, and infiltration of the surrounding muscle layer. Leiomyosarcoma has a smooth muscle immunophenotype, including smooth muscle actin, desmin, and caldesmon positivity (10, 11). Histone deacetylase 8 is also generally positive, and overexpression of p53 and P16 may also be detected (12). Ki67 staining indicates a high proliferative capacity in these tumors. Total hysterectomy remains the best initial treatment for patients with early ULMS (13). Ovariectomy and lymphadenectomy remain controversial for ULMS treatment. For patients with metastatic ULMS, resection of the metastatic tumor is also a feasible treatment strategy (14). Adjunctive therapy for ULMS includes chemotherapy and radiotherapy. Adriamycin and gemcitabine are optional drugs for patients with advanced or recurrent disease, with response rates of 17–36% (15, 16). Radiation therapy may be helpful for local recurrence.

When the volume of the abdominal tumor is high, the abdominal organs are significantly displaced and deformed, and their original anatomical positions and typical shapes are lost. The relationship between cancer and the abdominal organs becomes unclear, and the tumor’s location is not exact, thus making differential diagnosis difficult. CT 3D can track the vascular flow and its relationship with the tumor and display the vascular invasion around the tumor, including small vessels in any slice, and the main and variant arteries supplying blood to the abdominal tumor (17). Large abdominal vessels and their branches can also be reconstructed to observe the relationship between abdominal vessels and tumors, to improve the accuracy of locating and diagnosing abdominal tumors (18). The diagnostic resources available for the patient in our hospital are superior to those in other hospitals: preoperative CT 3D reconstruction was used to accurately evaluate the tumor’s size, relationship with surrounding organs and blood vessels, and tumor erosion range. The methods used were precise because of the accurate preoperative evaluation to ensure the success of the follow-up operation.

Abdominal neoplasms are rarely reported to extend from a blood vessel to the inferior vena cava. The most common tumor is renal cell carcinoma, but uterine, hepatocellular carcinoma, adrenal cortical carcinoma, and retroperitoneal sarcoma have also been reported (19). In our patient, the tumor invaded from the right ovarian vein to the inferior vena cava at the right renal hilar plane, reached the hepatic segment of the vena cava, and expanded intravascularly. This pattern of large-vessel involvement in ULMS is sporadic, and few studies in the literature have been reported (20, 21). In this case, the pelvic mass was large, the lesion range was wide, and the inferior vena cava was invaded, thus requiring difficult multidisciplinary operations. In addition to adequate blood preparation, the condition of the urinary tract, digestive tract, and cardiovascular system should also be fully assessed. If the mass is removed, the risk of tumor emboli shedding is high, and pulmonary and systemic circulation embolism, sudden death, and other serious complications can easily occur. Therefore, after multidisciplinary discussion, the corresponding departments were invited to complete the operation together, and single-drug chemotherapy with doxorubicin was administered after surgery.

In conclusion, ULMS with metastasis within the vena cava can be successfully treated by surgical resection. Patients should be assessed by a multidisciplinary team to determine the severity of the disease and to co-manage its complications so as ensure better clinical outcomes. Aggressive treatment at specialist centers and continued identification of new metastases may improve survival.

## Data availability statement

The raw data supporting the conclusions of this article will be made available by the authors without undue reservation.

## Ethics statement

The studies involving human participants were reviewed and approved by the Ethics Committee of Xinqiao Hospital, Army Medical University (Third Military Medical University). The patients/participants provided their written informed consent to participate in this study. Written informed consent was obtained from the individual(s) for the publication of any potentially identifiable images or data included in this article.

## Author contributions

LL and LiZ performed a literature search and drafted the manuscript. DY and YH acquired data. JY and LuZ contributed to the diagnosis and treatment. RX, XH and SL revised the manuscript. All authors read and approved the final manuscript.

## Conflict of interest

The authors declare that the research was conducted in the absence of any commercial or financial relationships that could be construed as a potential conflict of interest.

## Publisher’s note

All claims expressed in this article are solely those of the authors and do not necessarily represent those of their affiliated organizations, or those of the publisher, the editors and the reviewers. Any product that may be evaluated in this article, or claim that may be made by its manufacturer, is not guaranteed or endorsed by the publisher.

## Abbreviations

ULMS, Leiomyosarcoma of the uterus; CT 3D, Computed tomography three-dimensional; CTA, Computed tomography angiography; HE, Hematoxylin-Eosin; IHC, Immunohistochemical.
